# SMARTBATHROOM 102: STUDYING BATHING TRANSFER PERFORMANCE

**DOI:** 10.1093/geroni/igac059.1216

**Published:** 2022-12-20

**Authors:** Brian Jones, Jon Sanford, Peter Presti, Susan Lee, Sutanuka Bhattacharjya, Kyle Murphy

**Affiliations:** Georgia Institute of Technology, Atlanta, Georgia, United States; Georgia State University, Atlanta, Georgia, United States; Georgia Institute of Technology, Atlanta, Georgia, United States; Georgia State University, Atlanta, Georgia, United States; Georgia State University, Atlanta, Georgia, United States; Georgia Institute Of Technology, Atlanta, Georgia, United States

## Abstract

Age-related changes in functional ability, particularly among people aging with long-term disabilities, can impact safe performance of toilet and bathtub transfers and severely limit their ability to successfully age-in-place. The SmartBathroom is a state-of-the-art bathroom laboratory that features mechanically adjustable features and an array of sensors that can measure walking performance, location of feet and hands, and forces applied to the bathroom surfaces and fixtures. To complement the SmartToilet system, TechSAge recently developed the SmartBathing Transfer Testbed prototype that will enable us to study how different bathing environment configurations can impact transfer performance. The testbed consists of a height adjustable tub wall and three-wall, height and angle adjustable grab bars with integrated sensors to measure hand location and grip and load forces. This session will describe the SmartBathing Testbed and present results of studies with individuals both with and without ambulatory impairments performing simulated transfers within the testbed.

